# Modeling the metabolic profile of *Mytilus edulis* reveals molecular signatures linked to gonadal development, sex and environmental site

**DOI:** 10.1038/s41598-021-90494-y

**Published:** 2021-06-18

**Authors:** Jaanika Kronberg, Jonathan J. Byrne, Jeroen Jansen, Philipp Antczak, Adam Hines, John Bignell, Ioanna Katsiadaki, Mark R. Viant, Francesco Falciani

**Affiliations:** 1 Institute of Systems, Molecular and Integrative Biology, University of Liverpool, Liverpool, L69 3BX UK; 2 School of Biosciences, University of Birmingham, Birmingham, B15 2TT UK; 3 Radboud University, Nijmegen, The Netherlands; 4 Centre for Environment Fisheries and Aquaculture Science (Cefas), The North, Barrack Road, Weymouth, Dorset, DT4 8UB UK; 5 Estonian Genome Centre, Institute of Genomics, University of Tartu, Riia 23b, 51010 Tartu, Estonia

**Keywords:** Bioinformatics, Reverse engineering, Environmental sciences, Metabolomics

## Abstract

The monitoring of anthropogenic chemicals in the aquatic environment including their potential effects on aquatic organisms, is important for protecting life under water, a key sustainable development goal. In parallel with monitoring the concentrations of chemicals of concern, sentinel species are often used to investigate the biological effects of contaminants. Among these, bivalve molluscs such as mussels are filter-feeding and sessile, hence an excellent model system for measuring localized pollution. This study investigates the relationship between the metabolic state of the blue mussel (*Mytilus edulis*) and its physiology in different environments. We developed a computational model based on a reference site (relatively unpolluted) and integrated seasonal dynamics of metabolite relative concentrations with key physiological indicators and environmental parameters. The analysis of the model revealed that changes in metabolite levels during an annual cycle are influenced by water temperature and are linked to gonadal development. This work supports the importance of data-driven biology and its potential in environmental monitoring.

## Introduction

Anthropogenic pollution affects water quality and consequently represents a threat for ecosystem and human health (UN report, 2019). The increase in new chemicals released into aquatic ecosystems requires continuous development of monitoring techniques, especially concerning the detection of early biotic effects of chemical contamination. In the European Union, for the marine environment, this monitoring is performed under the Marine Water Framework Directive (MSFD)^1^. The MSFD aims to provide and support healthy marine ecosystems by establishing Good Environmental Status as set out by several environmental descriptors including, but not limited to, biological diversity, eutrophication, marine litter and anthropogenic contaminants.

Currently, chemical contaminant monitoring is undertaken by measuring levels of targeted contaminants in water, sediment and biota. In addition, sensitive indicator species are used as sentinels to assess ecosystem health through the use of biomarkers, thus providing additional information on the biological effects of environmental pollutants^2^. A few of these markers have been proven useful; for example vitellogenin presence in the plasma of male and juvenile fish is a robust biomarker of exposure to oestrogenic chemicals^3–5^. Molecular tools have been proposed as a sensitive approach capable of detecting organism-level effects very early on, with potential predictive power in determining a future deterioration of a populations fitness^6–9^. The advent of functional genomics technologies provides powerful tools that can be used to identify more complex molecular signatures that are predictive of toxicity^10–17^.

Mussels represent an excellent indicator species due to their wide ranging habitat, including both salt and freshwater, geographical distribution and their ability to filter vast amounts of water (e.g. *Mytilus edulis* can filter up to 15 ml water in a minute)^18–22^. Mussels have been used to monitor contaminants long before the advent of functional genomics technologies^21^. Whole organism biomarkers of toxic effects in mussels, such as scope for growth and stress on stress (i.e. time to death outside water)^23^ have been recommended by the International Council for the Exploration of the Sea (ICES)^24^. Mussel biomarkers exhibit a broad range of applications ranging from assessing the effects of UV filters^25^, urban wastewater^26^, oil pollution^27–30^, offshore gas platforms^31,32^ and a wide range of environmental pollution^20,33,34^.

More recently, mussel functional genomics has been applied to study their responses to tidal cycle, salinity, annual cycle, reproductive cycle and metal exposures^35–40^. While most ‘omics’ studies have relied on gene expression profiling, other omics technologies, such as metabolomics^13,41–44^ and proteomics^45–47^ have also been used and may provide a strongly phenotype-oriented view of the animal that aligns well with the objective of environmental effects monitoring.

Untangling the effects of multiple environmental stressors and identifying molecular mechanisms controlling organism physiology requires advanced computational methods resulting from the vast amount of data generated by these techniques. This study investigates the potential of systems biology to develop metabolism-based biomarkers interfacing changes in environmental parameters and whole organism biology. We approach this challenge by modelling the complexity of global metabolite changes of the blue mussel (*Mytilus edulis*) during a whole year and their relationship with location (site), temperature and salinity, gonadal stage, parasite load and the adipogranular tissue scoring index (ADG rate), an index from 0–4, describing the presence of ADG cells, as described in detail in^48^.

Mantle was used as this is the tissue in mussels where gonad forms and thus is highly relevant tissue for studying metabolic changes during the annual cycle in relation to geographical location, environmental and reproductive health parameters.These models identified a hierarchy of connected events that are linked to gonadal development. We show that the metabolic profiles can predict sex in the reference site. Ultimately, we provide evidence for the utility of this approach by showing that in more polluted waters, the metabolic state of males is altered and resembling this of females (feminisation).

## Methods

### Overview of the analysis strategy

**Figure 1:**
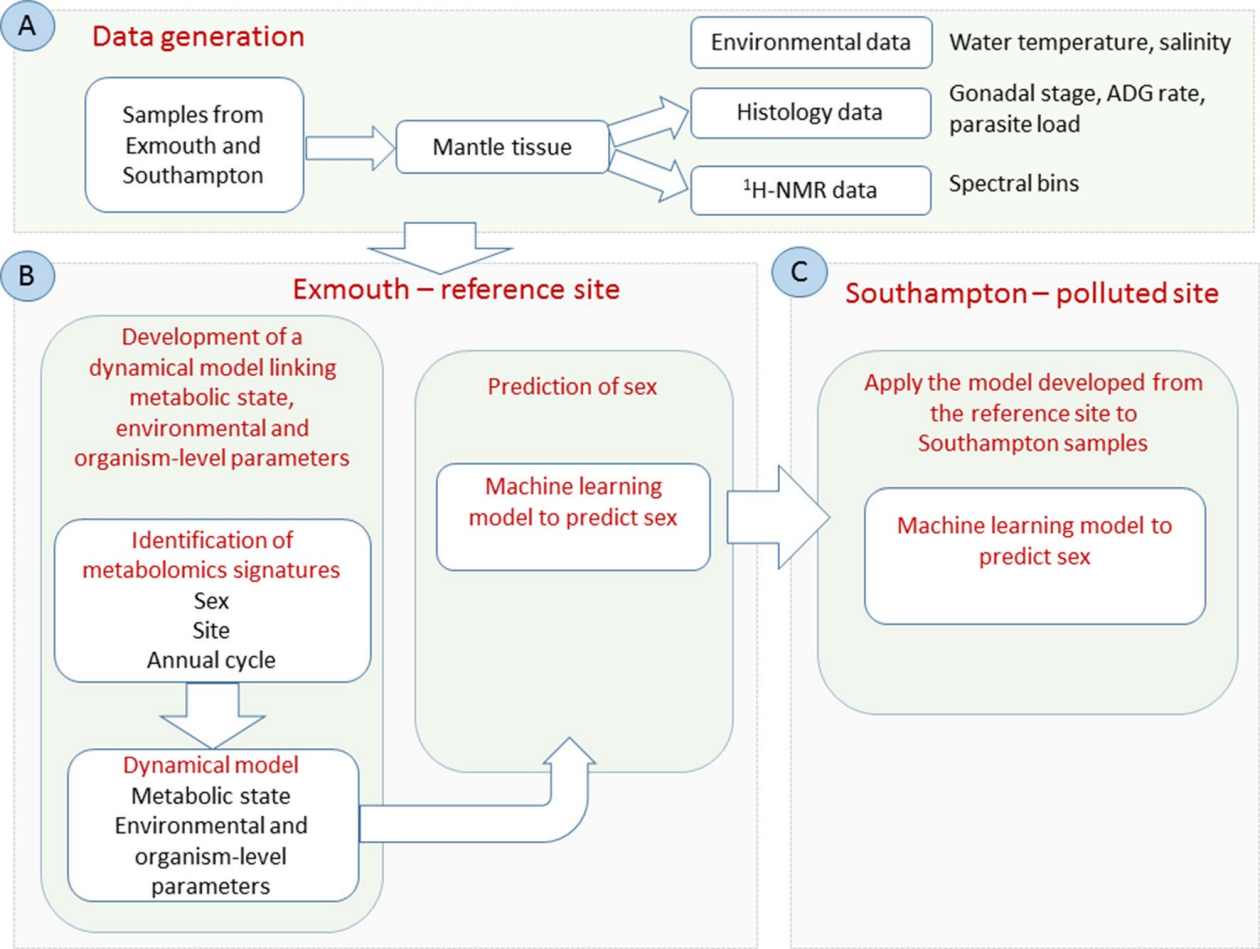
Overview of the data analysis strategy.

Figure 1 summarizes the overall structure of this study and the data analysis strategy. The first step (data production) involves the generation of a dataset representing the metabolic state of mussels sampled from Exmouth (a relatively unpolluted site, used as reference) and Southampton (a heavily industrialised site, used as polluted) over one year (Fig. 1A). During the data acquisition the mantle tissue is sampled to acquire both histological and ^1^H-NMR spectroscopy-based metabolomics data. Meanwhile, the environmental variables are collected. The second step (data analysis) involves the identification of metabolites linked to sex and reproductive stage (as determined by histopathology) in the Exmouth reference site, the development of a dynamical model representing annual variation, linking molecular changes to environmental and physiological parameters, and the development of a statistical model that can predict sex from the concentration of a few selected metabolites (Fig. 1B). In the third step, the application of the sex prediction model (developed for the reference site and applied to the Southampton site) revealed that the molecular state characterizing sex in mussels sampled from the Exmouth site is altered in organisms sampled from the more polluted site (Fig. 1C).

### Sample collection, determination of sex and physiological variables

A total of 50 mussels were collected from a rural reference site (Exmouth) and an industrial harbour site (Southampton), every month during one year^48^. Species, sex, gonadal development stage and the ADG rate were determined as previously described^48^. Gonadal status was defined as the degree of gonadal maturity scored on a scale between 0 and 5. The ADG scoring index, where 0 represents absence of ADG cells in vesicular connective tissue and 4 shows that majority of connective tissue volume are ADG cells.

Only individuals for which sex could be determined by histology were used for ^1^H-NMR metabolomics. In the exploratory analysis and in the model development, we concentrated on *M. edulis* only, as this species was present in both sites. However, due to low numbers of male *M. edulis* in Southampton, *M. galloprovincialis* and the hybrids of both species were included in the prediction of sex.

### NMR metabolomics analysis

NMR-based metabolomics analysis, including metabolite extraction, NMR analysis, data processing and metabolite annotation are described in Supplementary Methods.

### Statistical analysis: ANOVA and clustering

Metabolite bins were used for statistical analysis (ANOVA) and clustering with Hierarchical Ordered Partitioning and Collapsing Hybrid (HOPACH^49,50^). This clustering approach benefits from not having to know the number of clusters in advance and builds a hierarchical cluster from the data recursively, also allowing collapsing clusters at each level.

Using the larger dataset representing the full annual cycle developed with the reference site (Exmouth) samples, we first performed a two-factor ANOVA (as implemented in the data analysis software TMev^51^) and analysed the effects of time (12 months of field sampling) and sex for *Mytilus edulis*.

Since a smaller number of time points was available for the Southampton site a three-factor ANOVA (implemented in the statistical environment R) analysing the effects of time, sex and site (Exmouth and Southampton sites) was performed. In this ANOVA, time was factorized in three groups (April–May, June-July and December-January).

Spectral peaks with FDR-adjusted p-value (FDR) < 1% were considered significant. Data visualization of site-effects in clusters was performed on a more stringent statistical threshold to focus on the most significant results (FDR < 1e-8). For visualization purposes, the statistically significant metabolite bins identified by the first ANOVA were standardised (µ = 0, σ = 1). In order to reduce the complexity of the dataset we clustered significant metabolite spectral bins by using HOPACH^49,50^. The first round of clustering gave 7 clusters and was performed using the *abscos* similarity measure. Since this groups together negatively as well as positively correlated profile we performed an additional round of HOPACH clustering on cluster 3 that contained such heterogeneous profiles (Figure S1, Fig. 3).

### Principal component analysis

In order to visualize changes in the metabolic state of mussels across the annual cycle a principal component analysis (PCA), as implemented by the *prcomp* command in the Stats package in R^52^, was used. In short, three different PCAs were performed, (1) utilizing all significant metabolite bins, (2) using averages computed for each cluster, and (3) for specific subsets of clusters or metabolites. The reason for three different PCA analyses was to see whether data reduction by clustering preserves the annual cycle characteristics of the dataset and whether a small number of identified metabolites preserve the characteristics of the whole dataset.

To visualise the extent of the differences between the two sites, a PCA was generated based on the Exmouth samples only. Using the rotation matrix, which represent the calculated PCA parameters used to transform a dataset into principal components, the Southampton samples were projected into the same principal component space.

### Modelling the seasonal dynamics of mussel’s metabolic state

Dynamical models linking metabolite clusters, physiological measurements (gonadal stage, ADG rate, parasite load) and environmental parameters (salinity, water temperature) were built separately for male and female mussels in Exmouth using the algorithm TimeDelay ARACNE as implemented in the TDARACNE R package^53^. The procedure computes the mutual information between every combination of variables given a time delay. More precisely, for a given combination of features X and Y the algorithm computes the mutual information between datapoints of feature X_1..(P-delta)_ with feature Y_(1+delta)..(P)_, where P is the number of time-points and delta is the current delta value. This highest mutual information score across the different deltas is chosen and the delta value noted. Positive deltas then denote that feature Y is affecting feature X. Indirect connections are eliminated using the inequality principal (DPI). As our original dataset consisted of 12 time-points (12 months), we have used polynomial interpolation for each cluster of metabolites to get 120 time points (5th degree polynomial was used). In the TDARACNE models we choose a DPI tolerance of 0.15 and a time delay N = 20.

The models were represented using graphs where each node represented a cluster of highly correlated metabolites or one of the physiological or environmental measurements. Edges represented the strength of time-dependent relationship between variables. Nodes representing the metabolite clusters were color-coded to reflect the percentage of metabolite bins differentially expressed between Exmouth and Southampton.

### Predictive modelling

A Support Vector Machine (SVM) from R package Kernlab^54^ (linear kernel, C = 70, cross = 4) was used to develop a predictive model able to classify sex. To train the model, the Exmouth dataset was randomly split 5000 times into 75% training and 25% testing datasets. An SVM model was generated based on the significant metabolites for each split. Additional internal training/test splits were used to combat overtraining. The final model accuracy was determined by predicting the independent test dataset. All 5000 models were then used to predict sex for the Southampton samples (including additional samples from *Mytilus galloprovincialis* and hybrid of *M. galloprovincialis* and *M. edulis*). The resulting 5000 predictions for each sample were then averaged to define a representative sex prediction.

To confirm the visual inspection that GABA and ATP/ADP/AMP can discriminate sex in the winter in Exmouth but not Southampton, another KSVM model was built based on 2 metabolites (ATP/ADP/AMP and GABA) with linear kernel as before (C = 70, cross = 4), but only with cross-validation.

### Re-analysis of histological parameters

Previously published data^48^ representing the same mussels were re-analysed to test whether mussels in the Exmouth and Southampton sites were showing alterations in histological parameters that could be consistent with the predictions of our model. We tested whether the proportions of counts of each variable in the two sites are the same by using the prop.test function, followed by FDR-correction with *p.adjust* (method ‘*fdr*’), both within the statistical environment R^52^.

## Results

### *Mytilus edulis* mantle metabolic state changes in relation to seasonal cycle, sex and environmental location

We first tested the hypothesis that the metabolic state of the mussel mantle changes during the annual cycle, and that such variation reflects sex, reproductive stage and sampling site. We tested this hypothesis by identifying spectral bins associated with sex, time or site using a 3 factor ANOVA. From 1045 metabolic bins, there were 214 metabolite bins associated with sex, from which 133 metabolite bins did not demonstrate seasonal variability. More specifically, by using a two-factor ANOVA in the Exmouth site, we found that 79% of the spectral bins are changing significantly in at least one of the factors tested (825 out of 1045, FDR < 1%). Of these, 45% (474) were linked to time of the year, 30% (314) were changing both in time and between sex, and 37 were sex-specific but time-invariant (Figure S3). In comparing samples from the two sites we discovered 60% (628) site-linked spectral bins. From these site-linked bins, 273 were changing in time only, 38 were sex-specific, and 30 were changing in both time and sex (Figure S3).

### Sex-specific metabolites show different dynamics of seasonal variation in the two sampling sites

**Figure 2:**
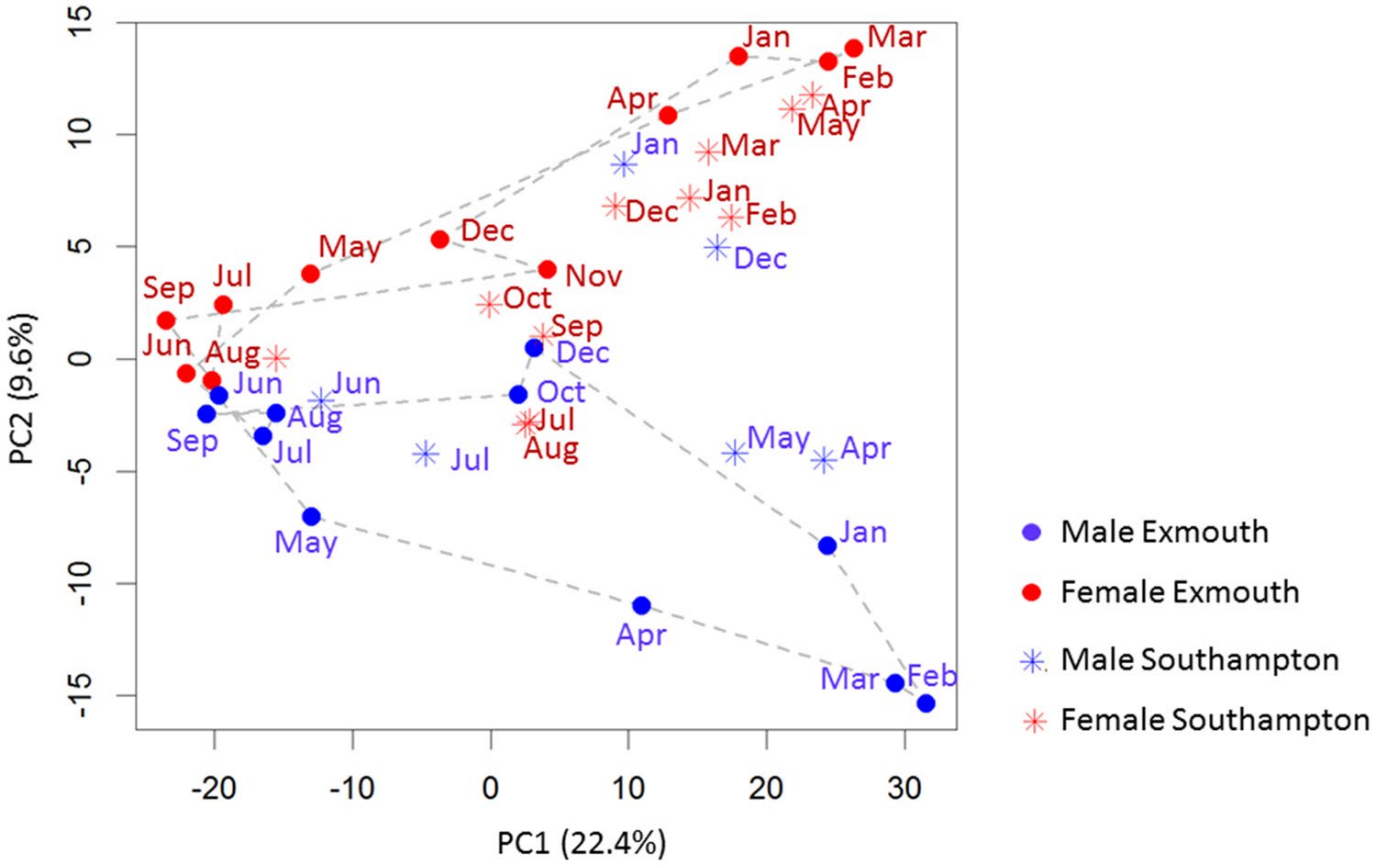
Principal component analysis (PCA) of cluster medians in both sex and sites during the annual cycle. Red represents females and blue males, solid dots are Exmouth (reference site) mussel medians, stars are Southampton medians.

Following confirmation that sex, site and time all affect the metabolic state of mussels, we asked whether the dynamics of metabolite changes are different in the two sampling sites. We first addressed this question by analysing the dynamics of change in the metabolic state of male and female mussels, in the two sampling sites, by using PCA. Figure 2 shows that male and female mussels from Exmouth are similar in the summer months, start diverging in the autumn, and reach maximum differentiation in late winter/early spring (February and March). Interestingly, while samples from the reference site show considerable separation between males and females in the period of peak gonadal development in late winter and early spring (Fig. 2), Southampton samples of male mussels collected in December and January are mixed with female samples, suggesting the existence of location-specific alterations in metabolism, linked to gonadal development (Fig. 2). More explicitly, we concluded that the metabolic profile of environmentally-sampled mussels follows a sex-specific trajectory across a year. We also concluded that the seasonal dynamics are affected in mussels sampled from the Southampton site to the extent that male mussels in winter show a very similar metabolic profile to the samples of female mussels from the reference site.

### Dynamical models, representing seasonal variation identify metabolite profiles linked to temperature, ADG rate and gonadal stage

**Figure 3:**
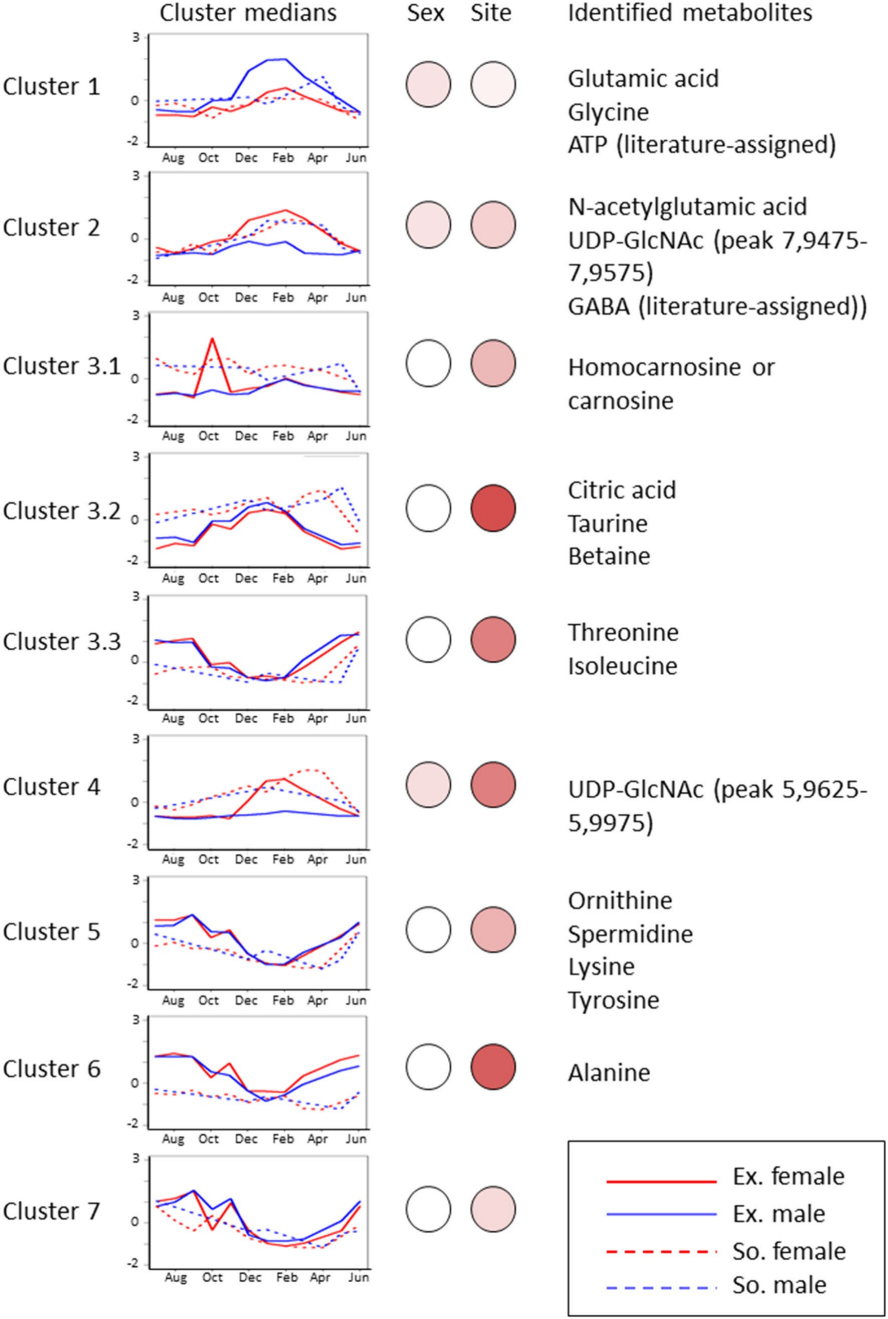
Clustering of metabolic profiles. Metabolite bins of Exmouth mussels have been clustered with HOPACH, a clustering tool that determines the number of clusters automatically. Cluster medians are used for visualization. The same Exmouth clusters are used to show cluster medians of Southampton samples. Female mussels are represented with red and males with blue, Exmouth with solid line and Southampton with dashed line. Percentage of significantly different (FDR 1e-8) bins in each cluster is shown by colour intensity. Putative metabolite identities are also shown.

While informative, the PCA provide a description of the general trends and does not represent explicitly the relationship between changes in the metabolome and gonadal stage during the annual reproductive cycle. We applied a computational method designed to learn the structure of a dynamical network from observational data to address this limitation. In order to reduce the complexity of the modelling task, we first set to reduce the number of variables (NMR spectral bins) to model. Clustering of metabolites changing over the whole year, revealed that the dynamics of the annual cycle can be described by 9 clusters (Fig. 3, Figure S1). Medians of clusters 1, 2, 3.1, 3.2 and 4 are positively correlated with gonadal stage, with maxima in the winter and negatively correlated with temperature (Spearman correlation, Table S3). Six clusters (3.1, 3.2, 3.3, 5, 6 and 7) are similar between male and female mussels, as shown in Fig. 3. Clusters correlated with gonadal stage (clusters 1, 2 and 4) show a stronger sex-specific response. PCA shows that cluster profiles are sufficient to capture the dynamics of the annual metabolic cycle (Figure S2), believed to be the first observation of its type for an aquatic species.

We then applied our computational approach to a dataset representing the median cluster profiles as well as relevant histological data (gonadal stage, count of ADG cells, parasite or disease burden) and environmental parameters (salinity and water temperature). We sought to use sex-specific models developed with this approach to identify molecular signatures that may be linked to gonadal development.

**Figure 4:**
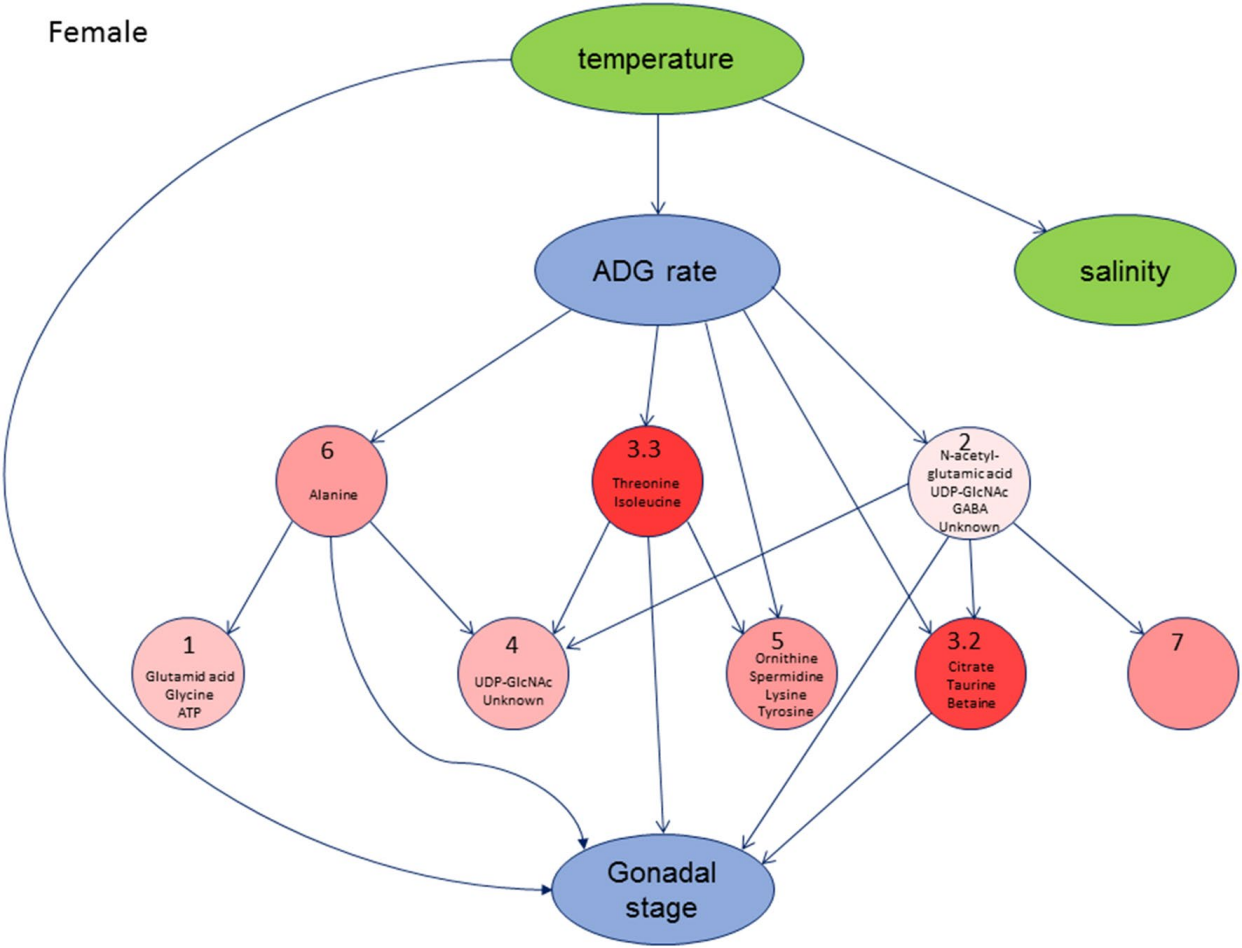
Dynamical TDARACNE model of the female mussels sampled from Exmouth (reference site). Red nodes represent metabolite clusters, green nodes environmental variables and blue nodes physiological variables. Intensity of red shows the percentage of metabolites significantly different (FDR 1e-8) between sites.

The model developed to represent seasonal dynamics in females (Fig. 4) places temperature as the most upstream node, directly connected to ADG rate and salinity. ADG rate further connects to a downstream layer of metabolite clusters. Interestingly, three metabolite nodes directly connect to gonadal stage which is the most downstream node in the network hierarchy of temporal events.

The model developed to represent male mussels (Figure S4) shows a different structure, from an initial visual inspection. However, similar to the female model, temperature is an upstream node. Cluster 3.3 is central in the network and downstream of ADG rate. Interestingly, in both male and female dynamical models, metabolite clusters 3.2 and 3.3 are directly upstream of gonadal stage and include the highest percentage of metabolites that are at different concentrations in mussels derived from the two sampling sites (Fig. 4, Figure S4).

### Development of sex-specific biomarkers

**Figure 5:**
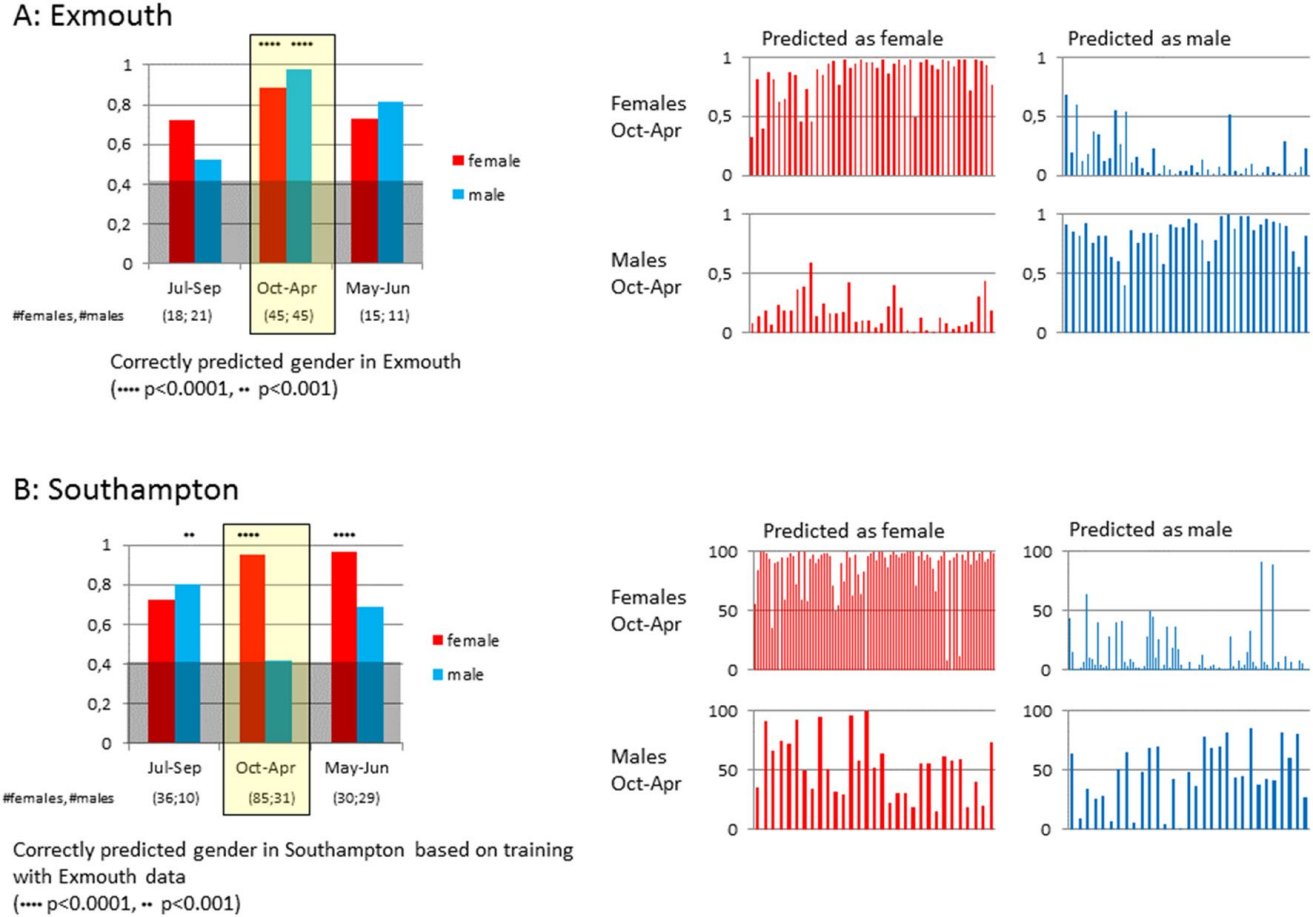
(**A**) Prediction accuracy of sex in Exmouth using support vector machine with 25% and 75% cross validation (left), and prediction of sex for individual mussels (right). (**B**) Prediction accuracies of Southampton mussels (left) and sex prediction for individual mussels in Southampton (right).

The analysis of the model described in Fig. 4 shows that metabolites present at different concentrations in mussels sampled from different geographical locations are upstream of gonadal development. Therefore, we reasoned that such metabolites may represent biochemical pathways that are sensitive to the different environmental conditions in the two locations and that are potentially directly or indirectly linked to gonadal development. If this is true, the levels of these metabolites could be informative of the mechanisms of sex differentiation in muscles and may also be sufficient to assign sex. We tested this by developing a statistical model that can predict sex from the metabolic state of mussels sampled from the reference Exmouth site (Fig. 5, Figure S5, and Figure S6). The model was very accurate in identifying male and female mussels based on their metabolic profile (97.8% accuracy) in the months between October and April. The model is still effective but less accurate at other times of the year. This is consistent with the timing of the development of the gonads (greatly reduced after spawning in late spring and summer). Metabolites contributing to the prediction were mostly mapped to clusters 1, 2 and 4 (Table S1), from which cluster 1 is mostly changing in males and not in females, and clusters 2 and 4 are female-specific, with no change in male mussels in the reference site. The top 20 metabolite bins predictive of sex are shown in Table S2 together with their putative identities.

We then tested whether the markers developed from the Exmouth dataset could be used to predict sex in the Southampton site. Only 41.9% of male mussels were predicted as “male” during the winter months (Fig. 5B left). Conversely, many of the male mussels were predicted as “female” with high probability (Fig. 5B right). Although in the Southampton site we have two different species and their hybrids we have observed that the model predictions were consistent, regardless of the species (Figure S5).

Some examples of individual metabolites that contribute to the sex-prediction model are shown in Figure S7. As expected, all of them show a differential concentration between males and females in the Exmouth site but not all of them in the Southampton site. We clustered these metabolites in 4 distinct groups on the basis of their sex and site profiles: (1) same sex-specific pattern between the two different sites (Figure S7 A: glycine, glutamate, unknown metabolites from cluster 4); (2) Different level of metabolite in the two sites but sex specific differences are in the same direction (Figure S7 B); (3) Different level of metabolite in the two sites and metabolites in Southampton males are similar to females in the Exmouth site (Figure S7 C: gamma-aminobutyric acid (GABA), ATP/ADP/AMP, UDP-GlcNAc, ornithine); (4) Different level of metabolite in the two sites and metabolites in Southampton females are similar to males in the Exmouth site (Figure S7 D: citrate). Notably, two metabolites that most contribute to sex prediction (GABA and ATP/ADP/AMP) are sufficient to separate males and females sampled from Exmouth in the winter (overall accuracy 90% with ksvm model) but fail to do so in the Southampton site (model accuracy 70% overall, but 82.4% for females and 25.8% for males) (Figure S8).

### Analysis of gonadal histology is consistent with model predictions

**Figure 6:**
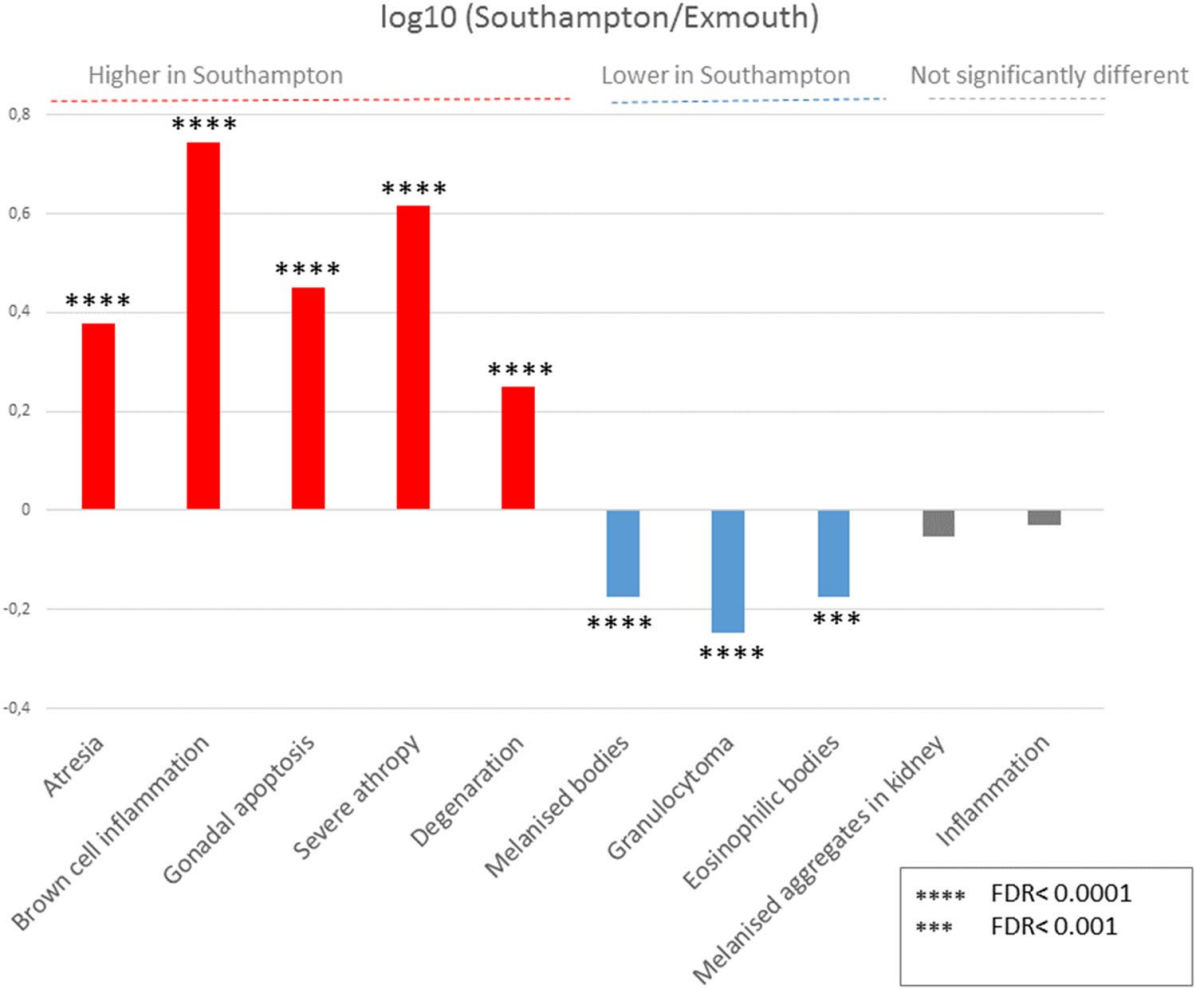
Ratio of histological parameters in Southampton and Exmouth mussels, based on re-analysed data from Bignell et al*.* 2008.

Our model is consistent with the hypothesis that gonadal development in mussels sampled from the Southampton site may be adversely affected. We reasoned that if this was true, we would expect a higher proportion of gonadal pathologies in mussels sampled from Southampton. We tested this hypothesis by analysing all histopathological parameters from Bignell et al.^48^. We could in fact verify that although there were no significant differences between sex ratios and the prevalence of intersex, other pathologies detected in whole mussel tissues (atresia, brown cell inflammation, severe atrophy, degeneration and gonadal apoptosis) were significantly more prevalent in the Southampton site (Figs. 6, Figure S9).

## Discussion

The most important finding of our study is the demonstration that the metabolic state of mussels sampled from the environment correlates with multiple biologically relevant parameters such as sex and gonadal development, and that it is potentially informative of site-specific environmental stressors, such as chemical contaminants. Our work provides useful knowledge to formulate hypothesis on the molecular basis of important biological processes such as gonadal development and may steer the development of future environmental monitoring tools.

### Are sex-specific metabolites important in sex determination?

The five metabolites that are most predictive of sex were glycine, glutamate, gamma-aminobutyric acid (GABA), ATP/ADP/AMP and UDP-GlcNAc.

Glycine has been previously shown to have higher levels in male mussels^55^. ATP/ADP/AMP is an energy currency of the cell and as it showed higher dynamics in male mussels and was correlated with gonadal development, it is possible that the sex-specificity is related to the content of ATP/ADP/AMP in sperm. This is supported by previous studies that relate ATP content with sperm quality^56^.

In addition to having different dynamics in male and female mussels, ATP/ADP/AMP was also affected by site. Interestingly, a previous ^1^H-NMR metabolomics study, which reported models predictive of the scope for growth, showed that pentachlorophenol (PCP, used as a pesticide), a chemical that uncouples oxidative phosphorylation, increases respiration rate and reduces scope for growth in lab-exposed mussels^13^. Moreover, using ^1^H-NMR spectroscopic data for adductor muscle for the same mussels as in the current study, the scope for growth was predicted to be lower in Southampton than in Exmouth^13^.

ATP concentrations have been shown to be inversely correlated with respiration rate in sea urchin^57^, meaning that site-effects might be due to specific chemicals which affect the oxidative phosphorylation, increase respiration rate and lower ATP concentrations. Unfortunately, we have no data available for the two sites about chemical contaminant concentrations.

Interestingly, gamma-aminobutyric acid (GABA) was linked to gonadal development in the model represented in Fig. 4 and ATP/ADP/AMP and UDP-GlcNAc were differentially abundant in the Southampton site.

Another interesting property of these metabolites is that three of them (glycine, GABA and glutamate) are known neurotransmitters (reviewed in 58, reviewed in 59, reviewed in 60). In addition to ATP being the ‘energy molecule’ in the cell and controlling a very large number of biological mechanisms, it also operates a wide range of channels involved in modulating the neuronal synapse and changes in its levels can have significant effects in nerve conduction (^61^, reviewed in ^62^). Since glycine, glutamate, GABA and ATP/ADP/AMP are all operating various ionotrophic receptors, it is reasonable to hypothesize that they may be involved in the control of sex determination or at least in the development and differentiation of the gonads.

There are several lines of evidence in support of this hypothesis. A recent gene expression study in the scallop has revealed that sodium- and chloride depending GABA transporters and sodium- and chloride dependent glycine transporters were over-expressed in the ovary^63^. Importantly, GABA has been shown to be present and functional in bivalves^64,65^. In oyster, the homolog of glutamic acid decarboxylase (cgCAD), the rate-limiting enzyme for conversion of glutamate into GABA, has been identified and shown to be functional^65^. The GABA transporter (GAT2) is also been found to be functional in oyster^64^.

These observations raise the question of what the role of GABA in the reproductive system of molluscs may be. Although there are no data in mussels, we know that in vertebrates, GABA regulates GnRH neurons^66^. Importantly, GnRH-like peptides play a role in the development of molluscan gonads as well. In the scallop, GnRH-like peptide has been found in the central nervous system where it stimulates spermatogenesis^67^. In vivo studies in scallop have showed that GnRH accelerates spermatogenesis in males while inhibiting oocyte development in females whereby shifting sex balance towards males^68^. GnRH is also involved in ovarian cell proliferation in abalone^69^. In the absence of a real understanding of sex determination and differentiation systems in mussels the possibility that GABA may also regulate GnRH neurons in molluscs represents an exciting hypothesis.

### Sex prediction models across geographical sites

We have shown that the metabolic state of male mussels sampled from the Southampton site is similar to females, in contrast to the well differentiated metabolic state of mussel samples from the Exmouth site. Despite the fact that *M. edulis* and *M. galloprovincialis* have different reproductive strategies^48^, we could see that the model based on the metabolomics data predicted that organisms sampled from Southampton were females, regardless of the species.

It is possible that chemicals in the water of the Southampton sampling site or other environmental and health factors, may be responsible for changes in the development of the mussel gonads that our model is detecting. This raises the potential that male individuals with the molecular state of a female in the more polluted site might show the detectable changes underlying complex pathological indicators of health, offering a new tool for environmental research. In fact, samples from the Southampton site show a significantly higher prevalence of gonadal pathologies, such as atresia and apoptosis as well as brown cell inflammation. Atresia has been shown to be associated with the presence of pollution and endocrine disrupting chemicals^70^, despite the fact that the molecular mechanism or role of these chemicals in mussels is not clear (reviewed in^71–73^). Moreover, it has been demonstrated that polluted water downstream of municipal effluents or on shipping routes can alter the male–female ratio^70,74^ or up-regulate vitellogenin^75^. Although the notion that the steroidogenic pathway is fully operational in invertebrate species (and its disruption leads to the above reproductive abnormalities) is highly controversial^71,72^, it is certain that more research is needed to characterise the basic physiology and endocrinology of reproduction in key marine ecological species such as mussels^76^.

## Conclusions

Our results show the potential of data-driven systems biology approaches using metabolomics for describing natural annual and reproductive cycles in wild species such as mussels. The most interesting feature of the models we developed is the fact that they identify metabolites with distinct seasonal dynamics that correlate with gonadal development. This led to the development of models that could predict sex from key metabolite markers and could also identify male mussels with female-like metabolic signatures in the more polluted site. We hypothesize that this signature might represent a molecular state associated with gonadal development, gonadal pathologies (atresia and apoptosis) and/or other general pathologies that we have identified to be more prevalent in the Southampton site. It is possible that environmental factors such as chemical pollution, food availability and/or physical–chemical water parameters in the Southampton site may be responsible for the perturbation of the metabolic signature we identified to correlate with sex.

We also show the potential of computational modelling of metabolomics data for generating experimentally testable hypothesis on the biology of mussels. We hypothesise a role for GABA in regulating the GnRH-like peptide in mussels. This intriguing hypothesis could be future tested by measuring GnRH levels after injection of GABA, or after GAT interference (similarly as in^64^ to study copper accumulation). The possible wider role of GABA in regulating other sex determination proteins could also be investigated by performing transcriptomics and/or proteomics experiments after GABA injection. Indeed, if this neurotransmitter induces changes related to gonadal development (either GnRH or other genes/proteins), it would also be possible to test which chemicals affect the levels of GABA. This could change the way we view mussels as sentinel species.

## Supplementary Information

Supplementary Information 1.

Supplementary Information 2.
