# Supplementary material for: Modeling the metabolic profile of Mytilus edulis reveals molecular signatures linked to gonadal development, sex and environmental site
Source: Sci Rep. 2021 Jun 18;11:12882. doi: 10.1038/s41598-021-90494-y (PMC8213754; doi:10.1038/s41598-021-90494-y)
Supplement: Supplementary file 1 — Supplementary Information 1. [file 41598_2021_90494_MOESM1_ESM.docx]

# Supplementary Methods

## NMR metabolomics analysis

*Metabolite extraction*: Following dissection and subsequent snap freezing in liquid nitrogen, polar metabolites were extracted from mussel tissues using a methanol:water:chloroform solvent system and a Precellys 24 homogeniser (Stretton Scientific, UK), as described previously (1). Each dried polar metabolite extract was resuspended in 650 μl of sodium phosphate buffer solution (0.1M, 90% H_2_O/10% D_2_O, pH 7.0) containing an internal chemical shift standard of 1 mM sodium 3-trimethylsilyl-2,2,3,3-d4-propionate (TMSP). 
*Data acquisition*: Samples were analysed using a Bruker Avance III 500 MHz NMR spectrometer operating at 500.18 MHz ^1^H resonance frequency, equipped with a 5 mm cryoprobe and BACS-60 automatic sample changer (Bruker Biospin, Coventry, UK). For each sample a two-dimensional ^1^H, ^1^H J-resolved (JRES) NMR spectrum was acquired using 16 transients per increment for 16 increments, collected into 16k data points, and spectral widths of 6009 Hz (12 ppm) in F2 (chemical shift axis) and 50 Hz in F1 (spin-spin coupling constant axis), with a 4.0-s relaxation delay. Datasets were zero-filled in F1 and both dimensions multiplied by sine-bell window functions prior to Fourier transformation. JRES spectra were tilted by 45º, symmetrized about F1, and then calibrated (TMSP, 0 ppm), all using TopSpin (Bruker). Data were exported as the 1-D skyline projections (along F2) of the JRES spectra (termed pJRES) (2). An example spectrum is shown in Figure S9.

*Pre-processing*: Each spectrum was binned between 10 and 0.2 with a bin width of 0.005ppm. Two regions were excluded (4.46 to 5.15 ppm, water; 7.6 to 7.76 ppm, residual chloroform from the extraction method). Data were normalized to total spectral area (TSA). Next, due to slight variation in the chemical shifts of some peaks, bins were compressed by calculating their mean. Six regions were compressed (7.11 to 7.16 ppm, 7.96 to 7.99 ppm, 7.99 to 8.02 ppm; 8.18 to 8.20 ppm, 8.26 to 8.29 ppm, and 8.58 to 8.61 ppm). A generalized log (Glog) transformation was performed with lambda = 3.75e-9 (3).

## Metabolite annotations

The annotations of the metabolites contributing most to each cluster were then determined using the web-based automated identification tool developed at the University of Birmingham (http://www.bml-nmr.org/). This tool makes use of a library of NMR spectra of ca. 200 pure metabolite standards (4). NMR chemical shift data from the literature (5,6) were also used to check the metabolite identities (**Table S1**).

# Supplementary Figures and Tables


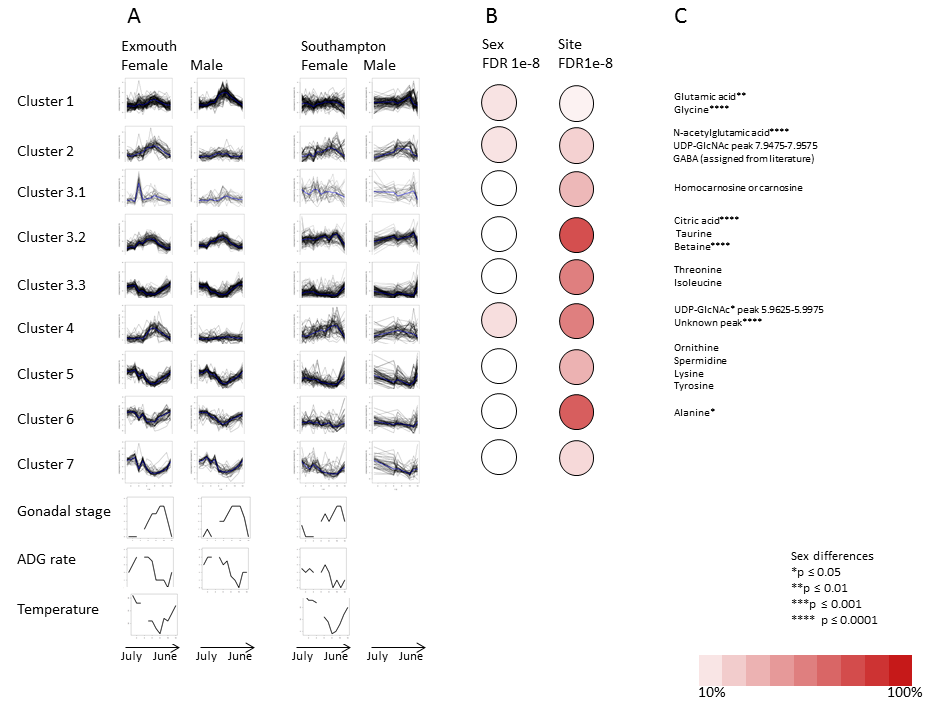


Figure.S 1. A: temporal profiles of female and male mussels in Exmouth and Southampton. B: percentage of metabolite bins in each cluster that are significantly different between male and female (left) and Exmouth and Southampton (right). C: identified metabolites in the clusters.


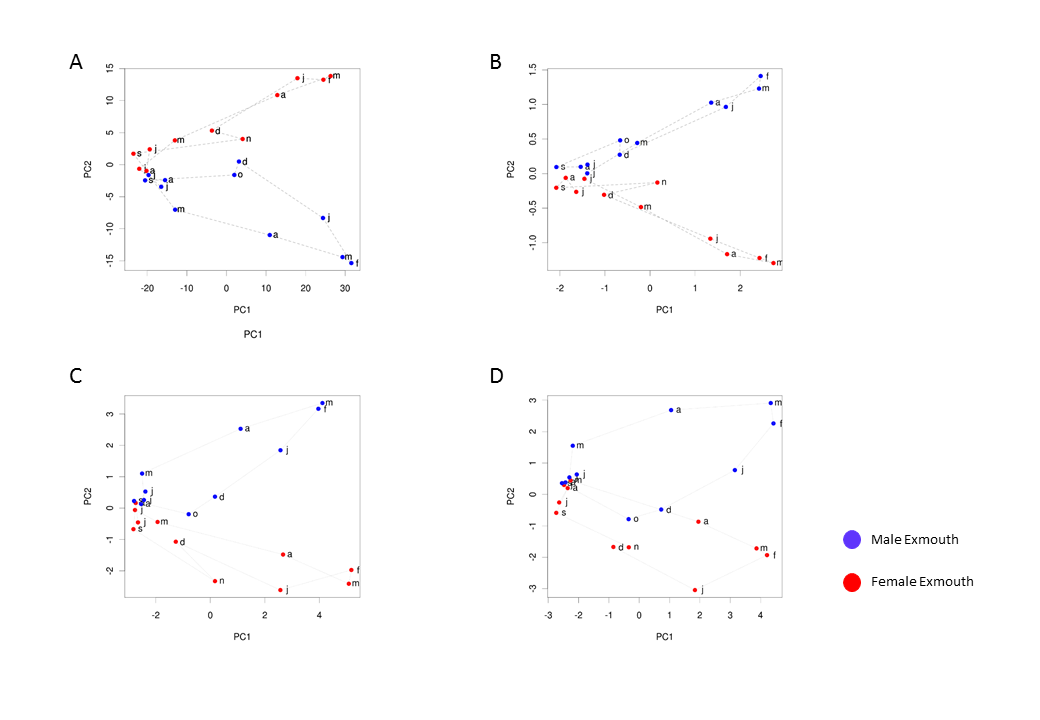


Figure.S 2. Principal component analysis (PCA) describing the annual cycle in the Exmouth site for male (blue) and female (red) Mytilus edulis. A: all significant metabolites; B: 9 cluster medians; C: 20 metabolite bins for the clusters (including all identified metabolites plus 5 significant peaks); D: only 15 identified metabolites


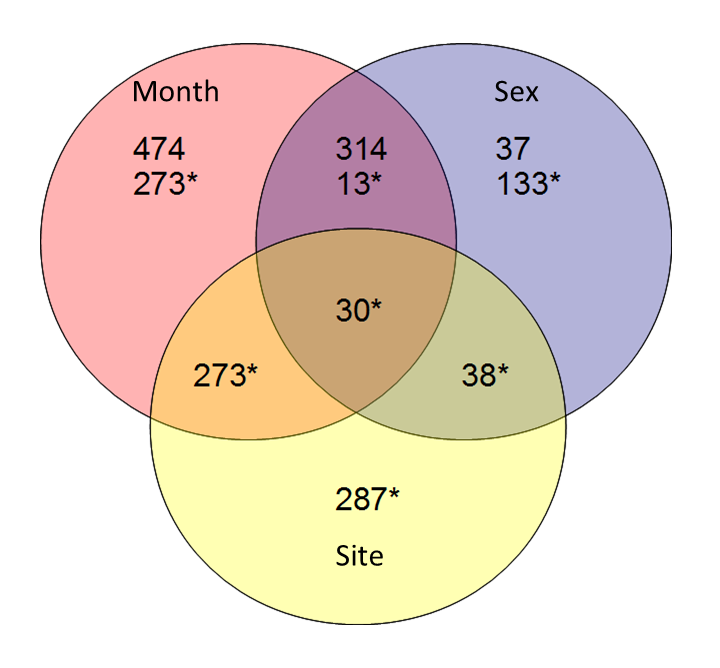


Figure.S 3. Figure S3. ANOVA for metabolites with FDR < 0.01 and their interactions. For site, ANOVA was performed for 3 groups due to lack of male samples in some months. For season and sex, tests were performed for 11 months

* Testing with 6 months in 3 groups only: Apr-May, Jun-Jul, Dec-Jan (FDR <0.01)


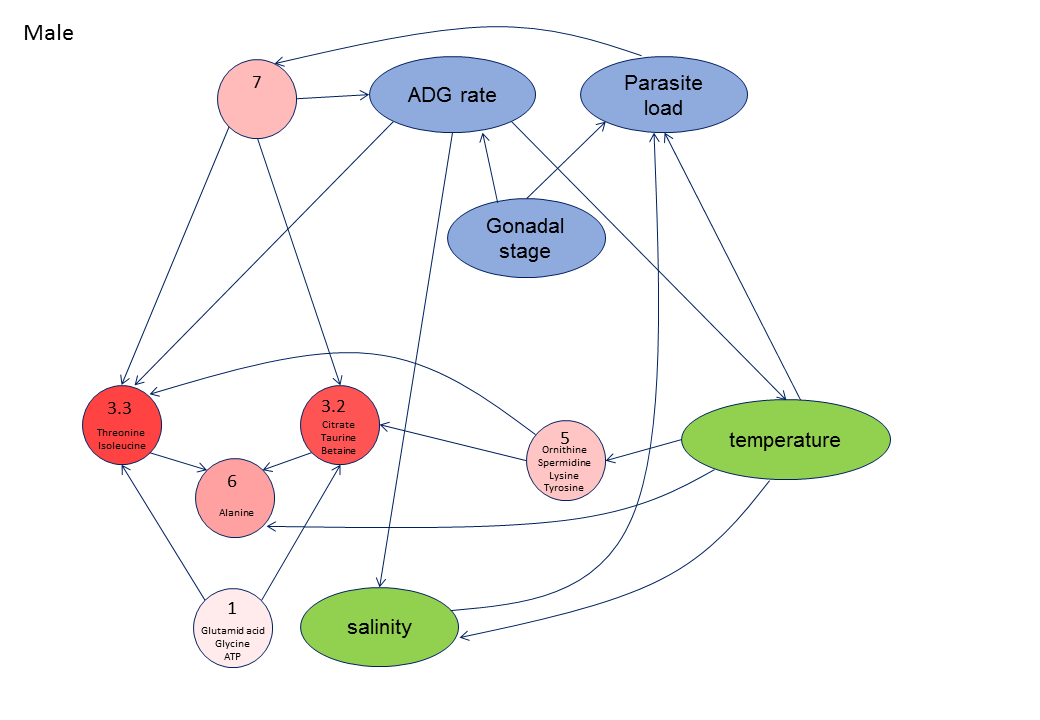


Figure.S 4. TDaracne network between clusters. Blue: physiological measurements, green: environmental measurements, red: metabolite levels. Intensity of red for metabolite levels indicate the percentage of metabolite bins significantly different between Exmouth and Southampton (male and female used for site differences)


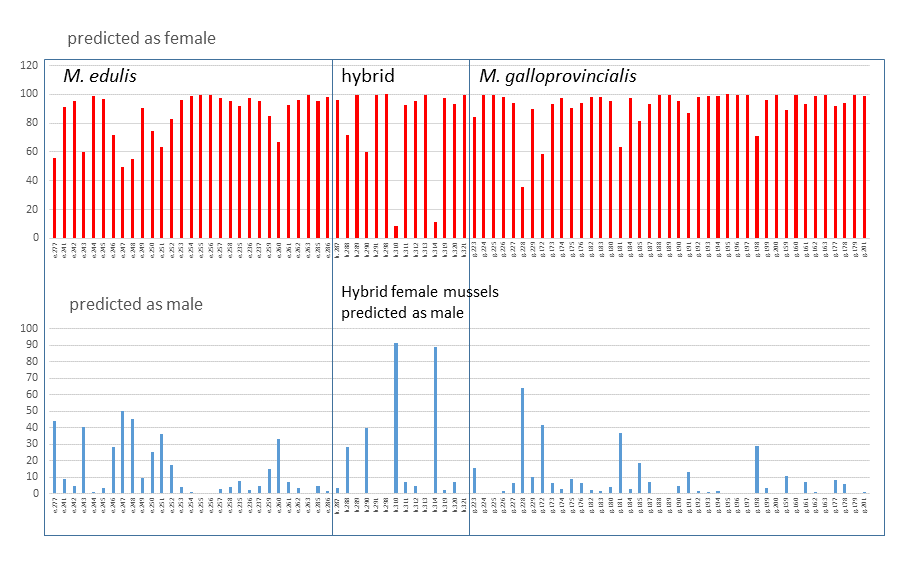


Figure.S 5. Sex predictions for female Southampton mussels for all species from October to April


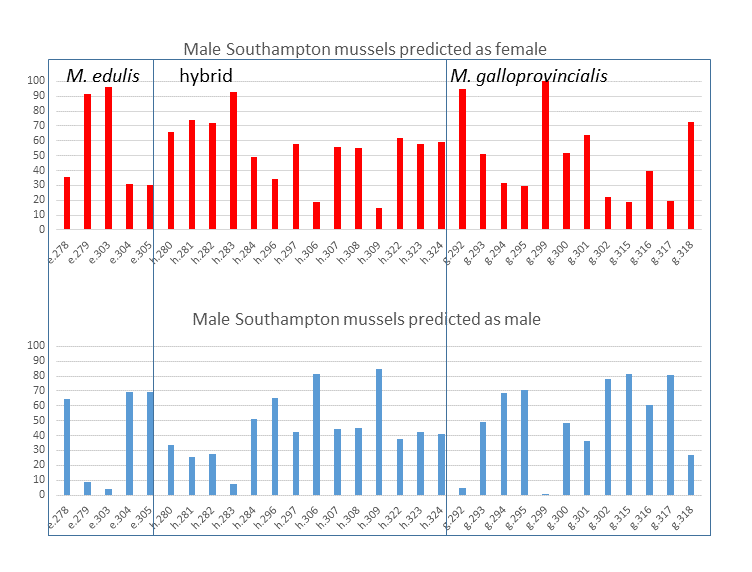


Figure.S 6. Sex predictions for male Southampton mussels in December and January for all species


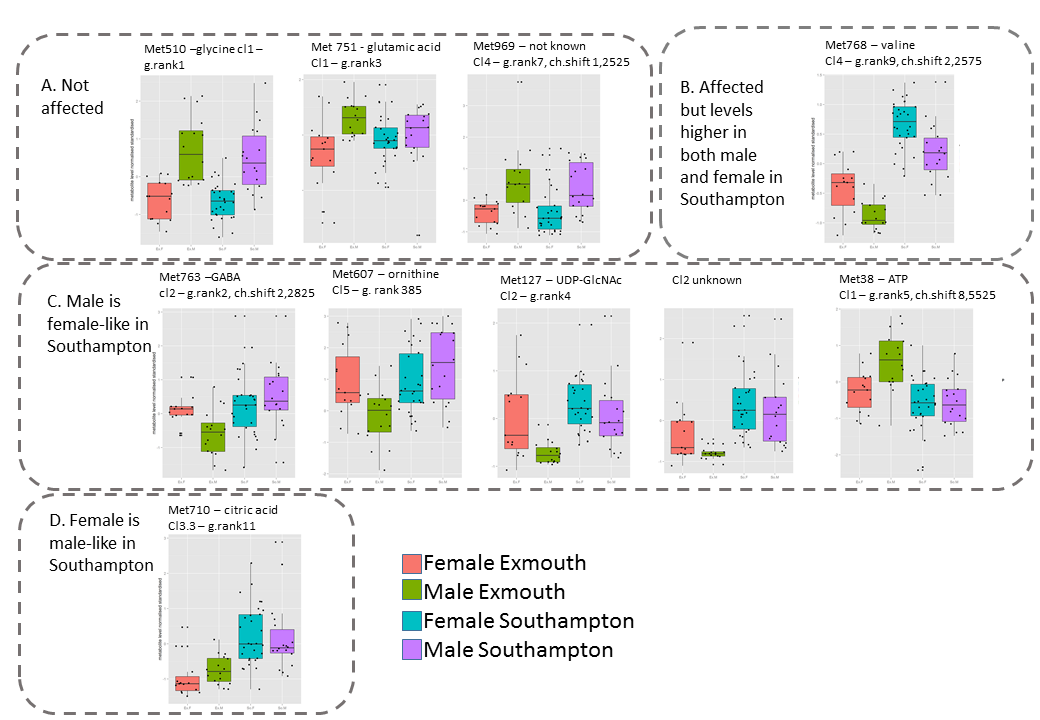


Figure.S 7. Metabolite levels in male and female mussels in two locations. A: Metabolites that differ between male and female mussels, but are not affected by site. B: Metabolites that differ between male and female mussels but are site-affected – male metabolite level in Southampton is more similar to the level of female mussels. C: Metabolite level in the female mussel is male-like in Southampton.


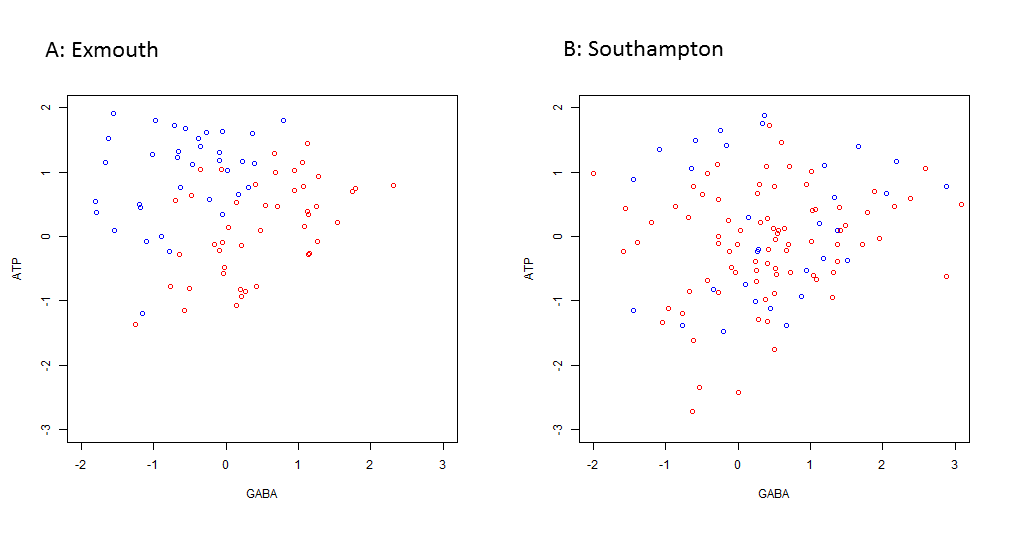


Figure.S 8. Discrimination of male (blue) and female (red) mussels by GABA and ATP in two locations: Exmouth (A) and Southampton (B).


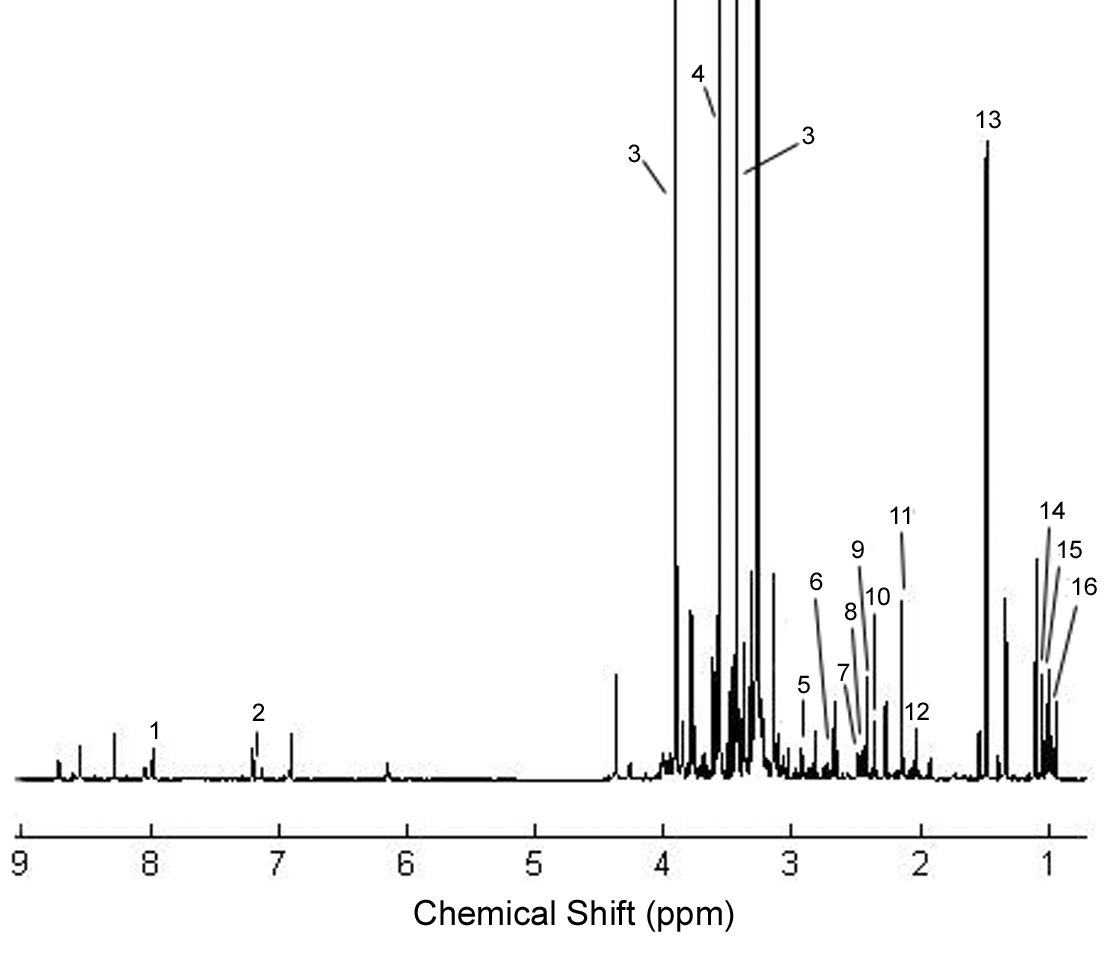


Figure S 9. An example of 1D projection of 2D JRES NMR spectrum from mantle tissue in M. edulis. Key: 1 – homarine, 2 – tryptophan, 3 – glycine-betaine, 4- glycine, 5 – hypotaurine, 6 – aspartate, 7 – glutamate, 8 – glutamine, 9 – succinate, 10 – acetoacetate, 11 – methionine, 12 – arginine/phosphoarginine, 13 - alanine, 14 – valine, 15 – leucine, 16 – isoleucine. Figure is from the PhD thesis of A. Hines (7)


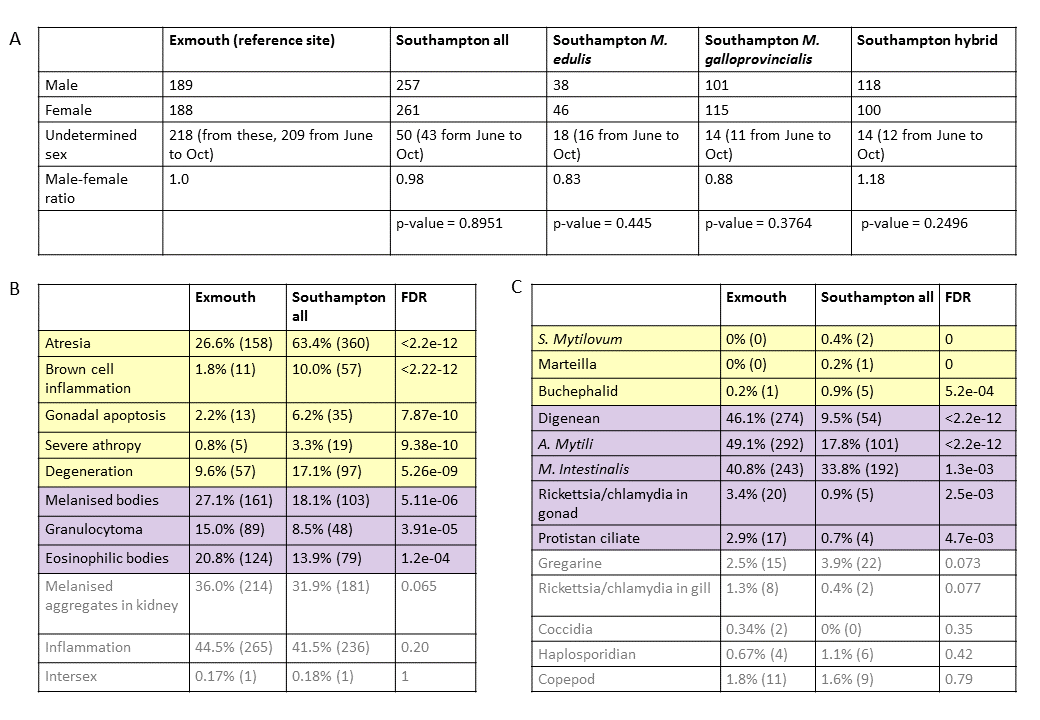


Figure.S10. A. Overview of sex from two sites. B. Morphological parameters summarised from Bignell et. al 2008: yellow background indicates variables that were detected significantly more often in the Southampton site and purple background indicates variables that are were detected significantly less often in the Southampton site. Gray font indicates variables that did not differ between sites. C. Presence of parasites summarised from Bignell et. al 2008: yellow background indicates parasites that were detected more often in the Southampton site and purple background indicates parameters that were detected significantly less often in the Southampton site. Gray font indicates parasites that did not differ in their presence between sites.

Table.S 1. GALGO was used to predict sex based on metabolite levels and the ranked sex-predicting metabolites were used in GSEA to see which clusters are enriched in sex-predicting metabolites.


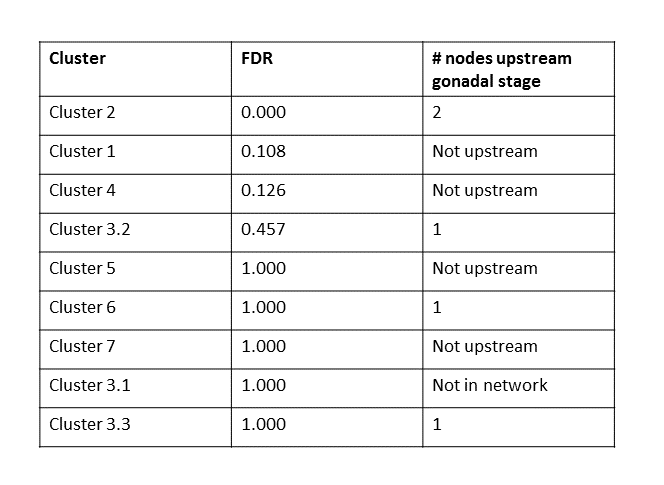


Table.S 2. Top 20 sex-specific metabolite bins and their putative identities from Birmingham Metabolite Library (<http://www.bml-nmr.org/>), Watanabe et al. 2015 (https://www.ncbi.nlm.nih.gov/pmc/articles/PMC4559106/pdf/11306_2015_Article_789.pdf) and Hurley-Sanders et al. 2015 (https://www.ncbi.nlm.nih.gov/pmc/articles/PMC4778454/pdf/cov023.pdf)


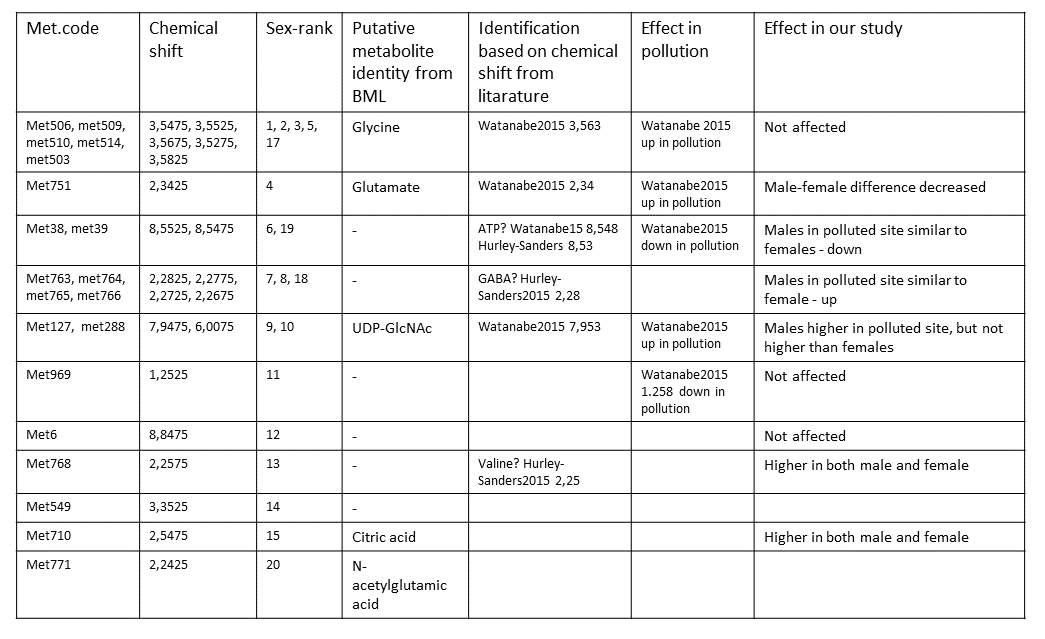


Table.S 3. Spearman correlation between physiological and environmental measurements and metabolite cluster medians in female (A) and male (B) mussels from Exmouth


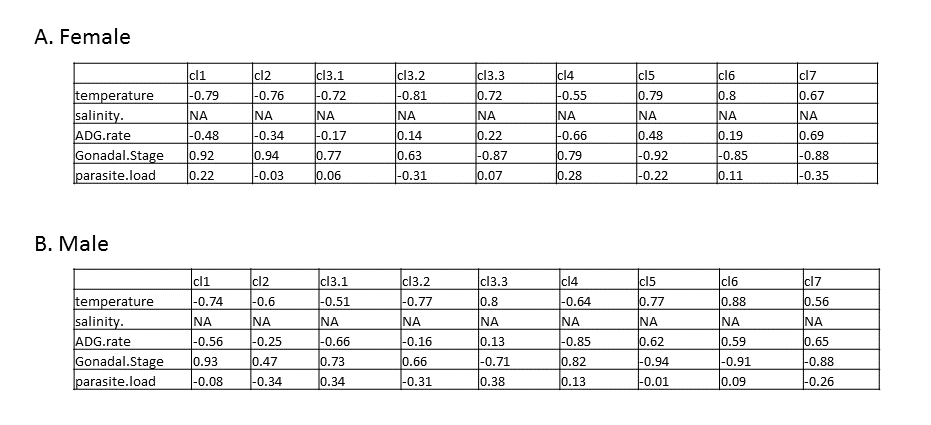


1. Wu H, Southam AD, Hines A, Viant MR. High-throughput tissue extraction protocol for NMR- and MS-based metabolomics. Anal Biochem. 2008;372(2):204–12.

2. Ludwig C, Viant MR. Two-dimensional J-resolved NMR spectroscopy: Review of a key methodology in the metabolomics toolbox. Phytochem Anal. 2010;21(1):22–32.

3. Parsons H, Viant M. Variance stabilising transformations for NMR metabolomics data. BMC Syst Biol. 2007;1(Suppl 1):22.

4. Ludwig C, Easton JM, Lodi A, Tiziani S, Manzoor SE, Southam AD, et al. Birmingham Metabolite Library: A publicly accessible database of 1-D1H and 2-D1H J-resolved NMR spectra of authentic metabolite standards (BML-NMR). Metabolomics. 2012;8(1):8–18.

5. Watanabe M, Meyer KA, Jackson TM, Schock TB, Johnson WE, Bearden DW. Application of NMR-based metabolomics for environmental assessment in the Great Lakes using zebra mussel ( Dreissena polymorpha ). Metabolomics. 2015;1302–15.

6. Hurley-sanders JL, Levine JF, Nelson SAC, Law JM, Showers WJ, Stoskopf MK. Key metabolites in tissue extracts of Elliptio complanata identified using 1 H nuclear magnetic resonance spectroscopy. 2015;3:1–10.

7. Hines A. The development and evaluation of NMR-based metabolomics as a tool for environmental monitoring using the common mussel. University of Birmingham; 2008.
