# Supplementary material for: Modeling the metabolic profile of Mytilus edulis reveals molecular signatures linked to gonadal development, sex and environmental site
Source: Sci Rep. 2021 Jun 18;11:12882. doi: 10.1038/s41598-021-90494-y (PMC8213754; doi:10.1038/s41598-021-90494-y)
Supplement: Supplementary file 2 — Supplementary Information 2. [file 41598_2021_90494_MOESM2_ESM.docx]

# SUPPORTING INFORMATION

Supplementary Methods: NMR extraction and metabolite annotation.

Figure S1. Alternative clustering visualisation

Figure S2. Principal component analysis across the annual cycle

Figure S3. Venn diagram of significant metabolites. In the Venn diagram, circles represent month, sex and site and numbers that are in each circle represent metabolite bins that were significant for each of the factors only, significant for 2 factors (numbers in the intersection of 2 circles) or for 3 factors (intersection of 3 circles). Numbers without the asterix show analyses with 2 factors (sex and month), numbers with the asterix show analyses with 3 factors (sex, month and site).

Figure S4. Model of male metabolite clusters, physiological and environmental measurements

Figure S5. Sex prediction for female mussels from Southampton

Figure S6. Sex prediction for male mussels from Southampton

Figure S7. Visualisation of individual metabolite levels in two locations

Figure S8. Scatter plot of GABA and ATP/ADP/AMP in two locations

Figure S9. Details of all re-analysed variables from Bignell *et al.* (48)

Table S1. GSEA of metabolite clusters

Table S2. Top 20 sex-specific metabolites and their putative identities

Table S3. Spearman correlations between physiological and environmental measurements and metabolite clusters medians
